# The concomitant of non-classical stereotactic body radiotherapy with tislelizumab based on multidisciplinary modalities for leiomyosarcoma: A case report

**DOI:** 10.1097/MD.0000000000040278

**Published:** 2024-11-08

**Authors:** Xiaona Han, Xiaotao Zhang, Yaru Lin, Jinying Li, Lan Yu, Li Liu, Wenzhi Tong, Xi Cheng, Xiao Li, Yanhao Liu

**Affiliations:** a Department of Radiation Oncology, Qingdao Central Hospital, University of Health and Rehabilitation Sciences, Qingdao, Shandong, China; b Medical Affairs, Beigene, Ltd., Beijing, China; c Cancer Prevention and Treatment Center, Qingdao Central Hospital, University of Health and Rehabilitation Sciences, Qingdao, Shandong, China.

**Keywords:** bone and soft tissue sarcoma, multidisciplinary modalities, radiobiology, radiophysics, stereotactic body radiotherapy, tislelizumab

## Abstract

**Background:**

Stereotactic body radiotherapy (SBRT) and immune checkpoint inhibitors (ICIs) could obtain a certain synergistic effect on bone and soft tissue sarcoma (BSTS). Given its low radiosensitivity, BSTS usually require an irradiation dose >65 Gy to achieve local control. Herein, we developed a non-classical SBRT technique called “onion-shaped simultaneous boost (OSB),” and reported a patient with prostatic leiomyosarcoma which received non-classical SBRT and other systemic treatments.

**Methods:**

In the case, a 49-year-old male patient was diagnosed with prostatic leiomyosarcoma in November 2018. In the radiotherapy plans, the maximum doses of the targets were more than 65 Gy, while the doses of the organs at risk (OARs) were strictly limited.

**Results:**

After receiving radiotherapy, ICI, and other multidisciplinary modalities, the patient achieved partial response (PR).

**Conclusion:**

In this clinical case, we have observed that the OSB technique, which employs an increased per-fraction radiotherapy dose without the need to match the single-fraction doses typical of conventional SBRT, effectively enhances the therapeutic impact on leiomyosarcoma without an increase in radiotherapy-related side effects. By integrating the OSB technique with a multidisciplinary array of antineoplastic strategies, we can more effectively manage sarcomas in clinical practice.

## 1. Introduction

Bone and soft tissue sarcomas (BSTS) are rare and heterogeneous malignant tumors that originate from mesenchymal tissue, and include fibrosarcoma, Ewing sarcoma, rhabdomyosarcoma, leiomyosarcoma, liposarcoma, pleomorphic sarcoma, and synovial sarcoma.^[1]^ Up to 50% of BSTS patients develop metastases in the course of the disease.^[2]^ Although radical resection is curable for patients with BSTS, a significant portion of BSTS was inoperable. The treatments for inoperable BSTS include chemotherapy, radiotherapy, tyrosine kinase inhibitors, and immune checkpoint inhibitors (ICIs). Some patients may be eligible to receive surgery in conjunction with preoperative or postoperative radiotherapy. Preoperative radiotherapy can enhance the rate of R0 resection, reduce the extent of surgery, and potentially convert non-resectable lesions into resectable ones. Postoperative radiotherapy, on the other hand, can decrease the rate of local recurrence. Recently, the feasibility, safety, and efficacy of stereotactic body radiotherapy (SBRT) for the treatment of various primary or metastatic carcinomas,^[3]^ including metastatic sarcoma, have been confirmed.^[4]^ Compared with conventional radiotherapy, SBRT has advantages of higher biological effective dose (BED), fewer fractions, and superior precision.^[5]^

Several ICIs targeted the tumor immune microenvironment toward the antitumor immunity context are being investigated in sarcomas. The preliminary clinical experience indicates that immunotherapy can be effective for refractory leiomyosarcomas.^[6]^ Tislelizumab is a novel anti-programmed death-1 (PD-1) monoclonal IgG4 antibody that being as an immunotherapeutic, anti-neoplastic drug. By blocking programmed death-ligand 1/2 (PD-L1/PD-L2)-associated cell signaling, tislelizumab could promote the production of cytokines and recover the cytotoxicity of T cells, thus leading to immune-associated tumor cell death.^[7]^ Currently, reports of tislelizumab in treatment for patients with BSTS remain rare.

SBRT combined with immunotherapy is a hot topic in anticancer research.^[3]^ The combination of SBRT with ICIs could remodel the anti-tumor immune balance and enhance the effect of radiation in vitro.^[8]^ Moreover, some studies suggested that the combination treatment of SBRT and PD-1 inhibitors is a promising strategy for patients with metastatic cancers.^[9]^ However, limited data exist regarding the safety and efficacy of SBRT combined with tislelizumab and other modalities for the treatment of metastatic BSTS. Based on classical SBRT, we have developed a unique technology called “onion-shaped simultaneous boost (OSB).” The fractions and single-fraction dose of OSB radiotherapy are between which of conventional radiotherapy and SBRT. Besides conventional targets, PTV and PGTV, OSB radiotherapy delivers higher irradiation doses to PGTVin which was within PGTV. Moreover, OSB radiotherapy has following core principles: the radiotherapy treatment plan strictly conforms to the dose limitation recommendations for organs at risk (OARs) as outlined in existing guidelines; the boost dose to PGTVin has no upper limitation, and the dose gradually increases from the periphery towards the core; the high-dose area to PGTVin does not encompass OARs. Theoretically, the hypoxic conditions within tumors lead to radiotherapy resistance, and an appropriately increased dose can enhance the therapeutic effect while ensuring safety. There is a scarcity of radiobiological data regarding this technique, which is the subject of our further experimental exploration. Currently, the method for calculating the biological effective dose for different radiotherapy fractionation schemes is still based on previous radiobiological animal and cellular experiments.^[10]^

The principle of SBRT involves the use of a linear accelerator to generate high-energy X-rays, employing non-coplanar arc techniques to irradiate the tumor target from various angles. This approach delivers a high dose to the central tumor lesion while rapidly decreasing the dose to surrounding areas, creating a steep dose gradient that protects adjacent normal tissues.^[11]^ The OSB technique leverages the characteristics of SBRT, utilizing the VARIAN EDGE 6MV X-ray system, high-precision high definition multi-leaf collimator, portal dosimetry for built-in field dose verification, and motion perfected control for fully automated device performance checks, along with cone beam computed tomography imaging technology, to achieve efficient and high-quality radiation therapy. This ensures a high dose gradient in the target area while reducing the dose received by organs at risk.

Leiomyosarcoma is a relatively common type of BSTS; however, primary prostatic leiomyosarcoma is exceedingly rare. For inoperable prostatic leiomyosarcoma, there is currently no standardized treatment approach. In this paper, we presented the outcomes of the case of refractory metastatic BST, who received OSB radiotherapy combined with tislelizumab and other multidisciplinary modalities.

## 2. Case Presentation

**Figure 1.**
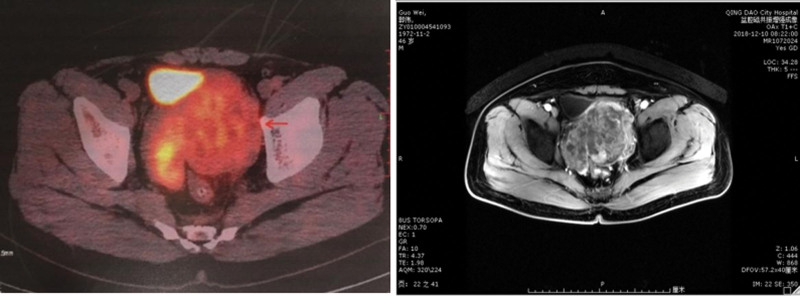
PET/CT and MRI of the primary tumor. CT = computed tomography, MRI = magnetic resonance imaging, PET = positron emission tomography.

**Figure 2.**
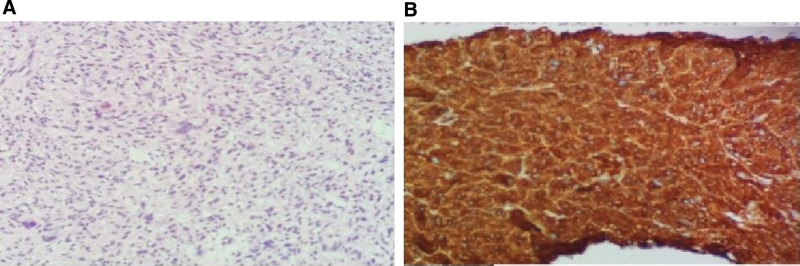
(A) HE staining (400×) and (B) immunohistochemistry SMA (200×) of prostate sarcoma. HE = Hematoxylin-eosin, SMA = smooth musclt actin.

A 49-year-old male who complained of frequent urination more than 20 times a day was diagnosed with prostatic leiomyosarcoma invading bladder and rectum, with multiple pelvic lymph nodes metastasis by PET/CT and MR on November 15, 2018 (Fig. 1). The pathological results of the biopsy suggested sarcomatoid (spindle cell) malignancy carcinoma. Immunohistochemical findings were SMA (+), CK (−), vimentin (+), P504S (−), PSA (−), S-100 (−), CD34 (−), PR (−) (Fig. 2).

**Figure 3.**
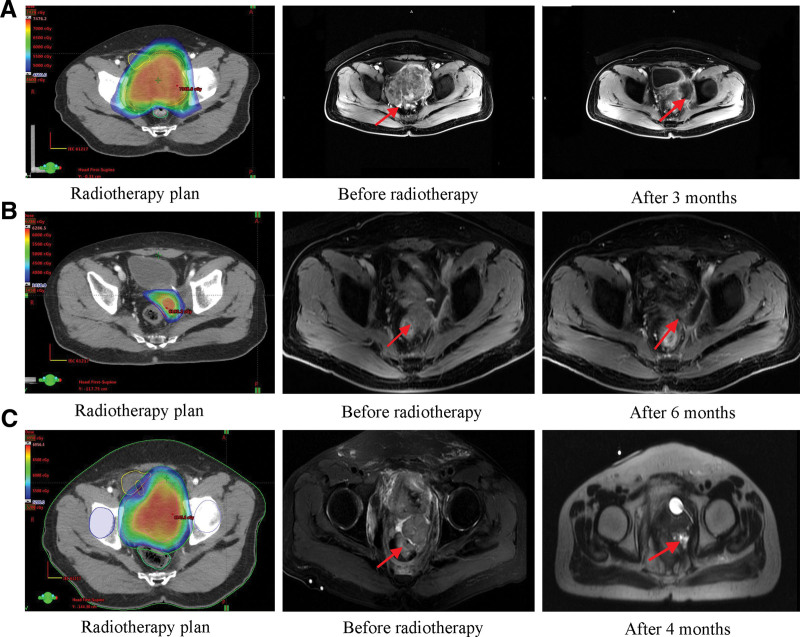
(A) Radiotherapy plan, MRI comparison of primary prostate lesion before radiotherapy and after radiotherapy for 3 months (achieved PR). (B) Radiotherapy plan, MRI comparison of local recurrent pelvic lesion before radiotherapy and after radiotherapy for 6 months (achieved PR). (C) Radiotherapy plan, MRI comparison of third local recurrent pelvic lesion before radiotherapy and after radiotherapy for 4 months (achieved PR). MRI = magnetic resonance imaging, PR = partial response.

After comprehensive evaluation, a multidisciplinary consultation was conducted. The multidisciplinary team thought this patient was not suitable for radical surgery, and suggested radiotherapy combined with systemic chemotherapy and anlotinib as first-line treatment. However, the patient refused chemotherapy. Anlotinib treatment began on December 18, 2018, and OSB radiotherapy for primary prostate lesion was conducted from December 24, 2018 to January 26, 2019. The prescription irradiation dose was as follows: 46 Gy in 23 fractions for 96% PGTV and 59.5 Gy in 17 fractions followed by 15 Gy in 6 fractions for 98% PGTVin (BED, 99Gy). The frequent urination was significantly relieved after radiotherapy, then the patient changed his tune to consent receiving chemotherapy. The chemotherapy of 30 mg liposomal doxorubicin every 3 weeks was conducted for 6 cycles from April 3 to July 18, 2019. The CT scan showed partial response (PR) on June 12, 2019. Following this, a urology consultation also ruled out the possibility of surgical resection of the residual lesion. PFS1 was 11 months (Fig. 3A).

**Figure 4.**
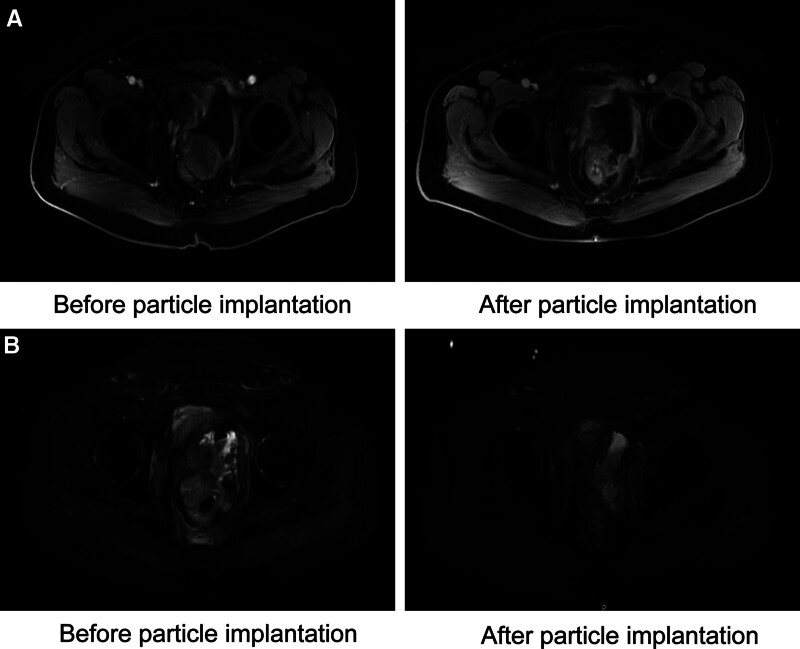
(A) MRI comparison of local recurrent pelvic lesion before I-125 seed implantation and after I-125 seed implantation for 2 months. (B) MRI comparison of second local recurrent pelvic lesion before I-125 seed implantation and after I-125 seed implantation for 3 months. I-125 = iodine-125, MRI = magnetic resonance imaging.

In October 2019, a local recurrence with rectal invasion was recognized by CT scan. Due to discharge of necrotic tumor and urethral obstruction, the patient underwent suprapubic cystostomy on November 25, 2019. Given the short interval time after OSB radiotherapy and the rectal invasion, the multidisciplinary team suggested brachytherapy instead of external-beam radiation therapy. The patient was treated by radioactive iodine-125 seed implantation (RSI) for the recurrent tumor in December 2019. The MR showed stable disease (SD; Fig. 4A). The patient stopped using anlotinib in January 2020 because of fistula hemorrhage. PFS2 was 3 months.

**Figure 5.**
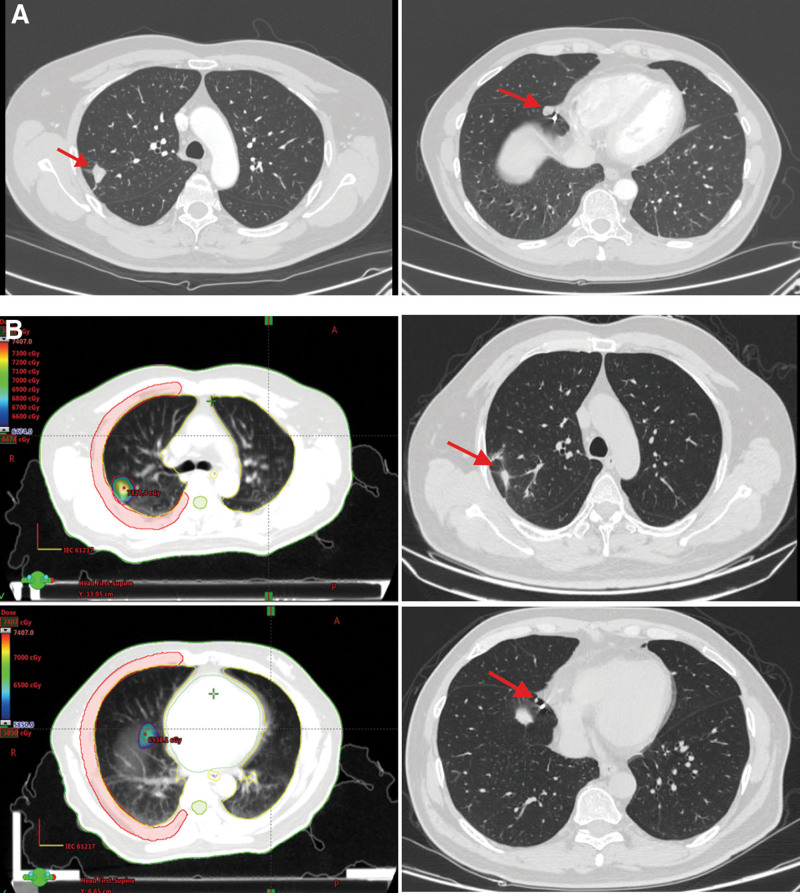
(A) CT of 2 lung metastasis. (B) Radiotherapy plan and CT after radiotherapy of 2 lung metastasis. CT = computed tomography.

CT scan showed 2 lung metastases on January 14, 2020 (Fig. 5A), and a radiofrequency ablation of lung metastases was conducted on February 21, 2020. On March 9, 2020, CT scan for the recurrent prostate lesion showed SD. From March 26 to July 3, 2020, the patient received radiotherapy for recurrent prostate lesion and lung metastases. In the OSB plan for recurrent lesion (Fig. 3B), the prescription dose was as follows: 34.5 Gy in 15 fractions for 96% PGTV (the BED was 42.4Gy) and 57 Gy in 15 fractions for 95% PGTV_in_ (the BED was 78.6 Gy). In the plan for 2 lung metastasis lesions (Fig. 5B), the prescription dose were as follows: 64.74 Gy in 13 fractions for 95% PTV1 (the BED was 96.98 Gy) and 58.5 Gy in 13 fractions for 95% PTV2 (the BED was 84.82 Gy). On October 21, 2020, the recurrent lesion and lung metastases achieved PR (Fig. 3B). PFS3 was 9 months.

On December 2020, a second recurrence occurred in the pelvis. The patient received tislelizumab 200 mg q3w for 3 cycles from December 2020, then underwent RSI again for pelvic metastases in January 2021 (Fig. 4B). However, a vesicorectal fistula developed and the patient underwent transverse colostomy on March 8, 2021. The recurrence lesion was slightly narrowed. PFS4 was 7 months.

He took Sulfatinib from April 7, CT showed organizing pneumonia On May 5, 2021, which was improved after anti-infection, hormone and other treatments.

**Figure 6.**
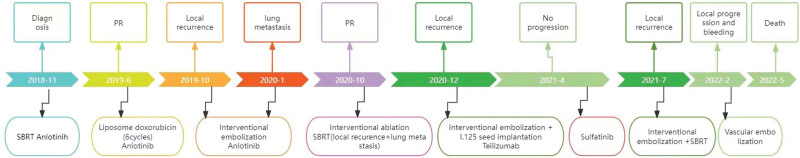
Treatment schedule.

A third recurrence occurred in the pelvis on July 2021. From September 17, 2021 to October 13, 2021, the third pelvic OSB radiotherapy for recurrent lesions was conducted. The prescription dose was as follows: 80Gy in 20 fractions for 95% GTV (the BED was 112Gy) and 60 Gy in 20 fractions for 95% PTV (the BED was 78Gy). CT scan showed PR on January 12, 2022 (Fig. 3C). The patient developed slowly with infection and bleeding in February 2022. Bilateral internal iliac arteriography plus embolization was performed on February 6, 2022 and March 29, 2022 because of massive hematuria. He died of massive hemorrhage on May 30, 2022. Figure 6 showed the timeline of the therapeutic effect.

## 3. Discussion

In recent years, some studies suggested SBRT plays an important role in the comprehensive treatment of sarcoma. Kubicek et al^[12]^ reported preoperative SBRT in 30 patients with soft tissue sarcoma resulted in improved local control rate. Sarcoma cells are characterized by low α/β value (0.5–5.4) among the cancers,^[13]^ which theoretically suggests that a higher dose per fraction is required to achieve local control. SBRT, as large-fraction radiotherapy, has changed the previous concept of radiobiology and further improved the tumor control rate.^[14]^ Although SBRT for BSTS has been reported in some studies,^[15–17]^ the non-classical SBRT, OSB technique, has not been previously reported. OSB radiotherapy can deliver higher BED than conventional radiotherapy to lesions which were unsuitable for SBRT, thus might achieve better efficacy, especially in cancers with low radiosensitivity.

BSTS may require a BED >65 Gy to obtain local control.^[18]^ In the present case report, OSB technique maximized the radiotherapy doses (>65 Gy) to the targets while limited the doses to OARs, and shortened the treatment time. The obvious feature of OSB technology is to change the uniform dose distribution in the target area required by the intensity-modulated radiation therapy and to maximize the radiotherapy dose in the target area, which is conducive to overcoming radiotherapy tolerance caused by hypoxia, improving biologically effective dose and ensuring the safety of organs.

ICIs targeting PD-1/PD-L1, including pembrolizumab, nivolumab, atezolizumab, and tiselizumab, has shown potent clinical efficacy for various cancers.^[19]^ Immunotherapy combined with SBRT was shown to produce synergistic effects. Meanwhile, PD-1 inhibitors could cover the deficiency of SBRT in unable to produce durable antitumor effects. Callaghan et al^[20]^ have confirmed the safety and efficacy of combining SBRT with concurrent PD-1 blockade in metastatic sarcoma. Chen et al^[21]^ reported the combination of SBRT and pembrolizumab achieved high local control rate in irradiated metastases from non-small-cell lung cancer. Le Guevelou et al^[22]^ have conducted a trial protocol of the concomitant SBRT with atezolizumab in soft tissue sarcomas to evaluate the efficacy, compared to SBRT alone. Our study firstly reported the clinical data of the synergistic effect of the concomitant of non-classical SBRT with tislelizumab on sarcomas.

Although the treatment regimen for BSTS has been improved, local recurrence and metastasis are still worthy of attention and affect the overall survival (OS) and prognosis of patients. The local recurrent rate after radical surgery ranged from 4% to 26%, and the prognosis of patients with recurrence was usually poor.^[23]^ Distant metastasis occurred in about 20% to 30% of BSTS patients, and the median OS time was about 12 months while the 5-year OS rate was only 10%.^[24]^

It still needs clarify that whether SBRT is superior to surgery and system treatment for metastatic sarcomas. In the case with prostate sarcoma, the patient received non-classical SBRT for the local recurrence and lung metastases and achieved PR, then received tislelizumab and other multidisciplinary modalities and achieved SD. The result indicated the non-classical SBRT combined with tislelizumab and other multidisciplinary modalities could control local recurrence and distant metastases, and might prolong the OS of BSTS patients.

## 4. Conclusion

In this clinical case, we have observed that the OSB technique, which employs an increased per-fraction radiotherapy dose without the need to match the single-fraction doses typical of conventional SBRT, effectively enhances the therapeutic impact on leiomyosarcoma. Importantly, this enhancement is achieved without an increase in radiotherapy-related side effects. By integrating the OSB technique with a multidisciplinary array of antineoplastic strategies, we can more effectively manage sarcomas in clinical practice. This integrated approach introduces a new horizon of hope for sarcoma treatment. At present, our technology still need further exploration in a larger sample size of patients, and we also look forward to the application of more unique and efficient radiotherapy technology in tumor treatment.

## Author contributions

**Conceptualization:** Xiaotao Zhang.

**Data curation:** Yaru Lin, Jinying Li, Lan Yu, Li Liu, Wenzhi Tong, Xi Cheng, Xiao Li, Yanhao Liu.

**Project administration:** Xiaotao Zhang.

**Resources:** Xiaona Han, Yanhao Liu.

**Writing – original draft:** Xiaona Han.

**Writing – review & editing:** Xiaona Han, Xiaotao Zhang, Yaru Lin, Jinying Li, Lan Yu, Li Liu, Wenzhi Tong, Xi Cheng, Xiao Li, Yanhao Liu.
